# A Retrospective Study on the Role of Tranexamic Acid in Reverse Total Shoulder Arthroplasty for Trauma Patients With Complex Proximal Humerus Fractures

**DOI:** 10.7759/cureus.78083

**Published:** 2025-01-27

**Authors:** Tirtha Rana, Hafiz Salman Mushtaq, Kashif Memon, Samuel Chan, Socrates Kalogrianitis

**Affiliations:** 1 Trauma and Orthopaedics, Queen Elizabeth Hospital Birmingham, Birmingham, GBR; 2 Trauma and Orthopaedics, Royal Derby Hospital, Derby, GBR

**Keywords:** complex proximal humerus fractures, open reduction internal fixation, reverse total shoulder arthroplasty, shoulder hemiarthroplasty, tranexamic acid

## Abstract

Background

The role of tranexamic acid (TXA) in primary elective hip, knee, and shoulder arthroplasty is well established. This is a retrospective study, which explores the efficacy of TXA in proximal humerus fractures (PHF) requiring shoulder arthroplasty.

Design and methods

Patients undergoing reverse total shoulder arthroplasty (RSA) for PHF between January 2022 and May 2024 in Queen Elizabeth Hospital (QEH), Birmingham, UK were identified. Patients were administered 1 g of intravenous TXA injection preoperatively during anesthetics induction. The parameters reviewed included changes in hemoglobin (Hb) levels from preoperative to postoperative, postoperative blood transfusion rates, and length of hospital stay.

Results

Out of 78 patients, 35 (45%) patients received TXA whereas 43 (55%) patients did not receive TXA preoperatively. No significant drop in Hb levels from preoperative to postoperative was observed (TXA: 1.7 ± 1.2 g/dL vs. non-TXA: 2.0 ± 1.3 g/dL, P = 0.30). Seven out of 78 (8.9%) patients required blood transfusion (3 (TXA) vs. 4 (non-TXA); 6 (86%) females vs. 1 (14%) males). In the blood transfusion cohort, patients from both groups required a longer length of hospital stay postoperatively (TXA: 20.3 + 16.0 days vs. non-TXA: 18.5 ± 14.8 days, P = 0.88).

Conclusion

Intravenous 1 g of TXA preoperatively was not associated with a significant decrease in postoperative Hb reduction in trauma patients undergoing RSA for PHF. Females undergoing RSA are at a greater risk of blood transfusion despite TXA administration. Future studies should consider investigating the dose-dependent efficacy of intravenous TXA on Hb drop postoperatively on trauma patients undergoing RSA.

## Introduction

A proximal humerus fracture (PHF) is a challenging problem in elderly patients. It significantly affects quality of life, including function and mobility, and patients may require hospitalization and rehabilitation [1,2]. It accounts for about 5% of all fractures and is one of the most common fractures in patients 65 years or older [3,4]. There is a higher prevalence of PHF in women (70%) with the highest frequency over the age of 85 years [5,6]. Medical, fracture-specific, and associated injuries, along with functional complexities, make the treatment of this condition challenging [7].

Around 67% to 85% of PHFs are treated conservatively without surgery [8]. Surgical options for PHF are dependent on fracture pattern, pre-injury function, patient anatomy, pre-existing medical conditions, selection bias, and surgeon skill level [7]. The role of open reduction internal fixation (ORIF) has remained stable, hemiarthroplasties have declined, and reverse total shoulder arthroplasty (RSA) has increased over the past decade ranging from 26% in 2011 to 67.4% in 2015 [7,9,10]. 

Complications of shoulder arthroplasty include hematoma formation, blood loss, and wound infection [11,12]. Significant blood loss postoperatively in patients can sometimes require blood transfusion. Blood transfusion comes with a host of complications including the risk of immunogenic reactions, bacterial infections, wound inflammation, and respiratory tract infections [13]. Blood transfusion following shoulder arthroplasty has been associated with a greater risk of sepsis, respiratory infection, venous thromboembolism, and periprosthetic joint infection [14]. Improving perioperative blood loss management would aid in reducing the rate of blood transfusion which in turn could lessen the risk of post-transfusion infection.

Tranexamic acid (TXA) is commonly used in lower-limb arthroplasty to decrease perioperative blood loss and transfusion rates [15]. TXA is an anti-fibrinolytic agent that competitively binds to the lysin-binding sites on plasminogen [16]. This mechanism of action by TXA prevents fibrinolysis stabilizing blood clots, which consequently reduces active bleeding [17]. Although it is a common practice to use TXA preoperatively in hip and knee arthroplasty, TXA administration in shoulder arthroplasty is dependent on the preferences of the surgeon performing the operation. Evidence shows that TXA reduces blood loss and drain output in elective shoulder arthroplasty [18,19,20]. Our study aims to look into the efficacy of TXA in patients undergoing shoulder arthroplasty for complex PHF by investigating the change in hemoglobin (Hb) levels from preoperative to postoperative and the requirements for blood transfusion.

This article's findings were previously presented at the British Pakistani Orthopaedic Society Conference held in Birmingham, United Kingdom, on 21st April 2024, and in the British Indian Orthopaedic Society Conference held in Newcastle, United Kingdom, on 6th July 2024.

## Materials and methods

Study design and patient enrolment

This was a retrospective study involving a total of 78 patients who underwent RSA for PHF. All patients identified for this study were inpatients at Queen Elizabeth Hospital (QEH), Birmingham, United Kingdom. QEH is a major trauma center receiving complex trauma patients from the UK nationwide. Shoulder replacements were performed by four senior orthopedic surgeons specializing in shoulder surgeries (KM, SC, SK, and ES). The procedure was performed with the patient under general anesthesia with an interscalene block. Intravenous injection of 1 g TXA was administered during general anesthetics induction on the preference of the surgeon performing shoulder arthroplasty.

Data collection

All patients who underwent shoulder arthroplasty at QEH between January 2022 and May 2024 were identified. Electronic medical records of patients were collected through PICS (participant identification centers), software used at QEH. The last Hb level recorded prior to arthroplasty was considered as preoperative Hb. Similarly, Hb levels on postoperative day 1 were considered to be postoperative Hb. Hb changes were considered to be the difference in Hb levels from preoperative to postoperative. Information on the dose, route, and administration of TXA was collected through paper anesthetic charts for all surgeries and/or TXA prescribed on PICS.

The inclusion criteria were any patient undergoing RSA for PHF. The exclusion criteria were shoulder arthroplasty for shoulder dislocation, revision RSA for shoulder dislocation, ipsilateral multiple fractures, preoperative blood transfusions, and shoulder arthroplasties including TSA or hemiarthroplasty.

Data on postoperative blood transfusion was collected via accessing electronic records of the administration of red cells prescribed on PICS. Medical notes on PICS were identified whereby the decision to transfuse red cells was made by the doctors looking after the patient in the Trauma and Orthopaedics ward at QEHB. The Hb threshold for blood transfusion was Hb at or below 7.0 g/dL and/or as per senior doctors’ clinical judgment on the patient’s symptoms of postoperative anemia.

Statistical analysis

Statistical analysis was carried out using the statistical software package, IBM SPSS (statistical package for the social sciences), version 29.0.2.0 (IBM Corp., Armonk, NY, USA). Data was tested for normal distribution using the Kolmogorov-Smirnov test. Two-tailed student t-test or Mann Whitney U test where appropriate was performed to evaluate the differences between the TXA group and the non-TXA group. A P-value of <0.05 was considered to be statistically significant.

## Results

Out of a total of 78 patients included in the study, 61 (78%) were female patients and 17 (22%) were male patients. The mean age was 75.1 ± 10.1 years, ranging from 35 to 94 years of age. The average preoperative Hb was 11.7 ± 1.5 g/dL and the average postoperative Hb was 9.8 ± 1.3 g/dL. The average reduction in Hb postoperatively was 1.9 ± 1.3 g/dL (Table 1).

**Table 1:** Patient demographics and total perioperative hemodynamics

Patient characteristics	
Total no of patients	n=78
Sex female/male, N (%)	61/17
Mean age (SD)	75.1 (10.1)
Mean pre-op Hb (SD)	11.7 (1.5)
Mean POD1 Hb (SD)	9.8 (1.3)
Mean Hb drop (SD)	1.9 (1.3)
Mean length of postoperative hospital stay (SD)	7.8 (8.8)
Mean length of total inpatient hospital stay (SD)	13.4 (12.1)
Hb = hemoglobin, SD = standard deviation, POD = postoperative day	

Of 78 patients in this study, 35 patients (45%) had received TXA preoperatively whereas 43 patients (55%) had not received any TXA preoperatively (control group). The average age was comparable between the two groups with 75.9 ± 8.8 years in the non-TXA group and 74.1 ± 11.7 years in the TXA group. The TXA group comprised 26 (74%) females and nine (26%) males whereas the non-TXA group consisted of 35 (81%) females and eight (19%) males. The average preoperative Hb was 11.9 ± 1.6 g/dL in the TXA group compared to the non-TXA group with 11.6 ± 1.4 g/dL (P < 0.38). There was a significant difference in the postoperative Hb group between TXA and non-TXA groups (10.2 ± 1.4 g/dL vs. 9.5 ± 1.1 g/dL, P = 0.05). There was no statistical difference between the two groups in levels of Hb drop from preoperative to postoperative (TXA group: 1.7 ± 1.2 g/dL vs. non-TXA group: 2.0 ± 1.3 g/dL, P = 0.30). Our results did not identify a significant drop in Hb levels postoperatively between the two groups (Table 2).

**Table 2:** Patient hemodynamics between groups

Patient characteristics			
	Non-TXA (n=43)	TXA (n=35)	P-value
Sex female/male, N (%)	35/8 (81/19)	26/9 (74/26)	
Mean age (SD)	75.9 (8.8)	74.1 (11.7)	0.44
Mean Pre-op Hb (SD)	11.6 (1.4)	11.9 (1.6)	0.38
Mean POD1 Hb (SD)	9.5 (1.1)	10.2 (1.4)	0.05
Mean Hb drop	2.0 (1.3)	1.7 (1.2)	0.30
Mean length of postoperative hospital stay (SD)	7.9 (8.6)	7.7 (9.3)	0.86*
Mean length of total hospital stay (SD)	14.1 (12.7)	12.7 (11.5)	0.73*
Hb = hemoglobin, SD = standard deviation, POD = postoperative day. P-value using two-tailed t-test. *Mann Whitney U test.			

Blood transfusions

Seven out of 78 patients (8.9%) in this study required blood transfusion postoperatively, with six (85.8%) females and one (14.2%) male. Out of seven patients requiring postoperative blood transfusion, three patients had received TXA preoperatively whereas four patients had not received TXA preoperatively. The average age was comparable between the groups, with 80.5 ± 1.9 years in the non-TXA group and 76.3 ± 3.5 years in the TXA group. Patients from the non-TXA group who required blood transfusion had an average preoperative and postoperative Hb of 10.7 ± 0.3 g/dL and 7.9 ± 0.3 g/dL, respectively. On the other hand, patients from the TXA group receiving blood transfusion had an average preoperative and postoperative Hb of 10.3 ± 1.3 g/dL and 8.7 ± 2.2 g/dL respectively. The Hb drop between the two groups was an average of 2.8 ± 0.0 g/dL in non-TXA vs. 1.7 ± 2.3 g/dL in the TXA group. The total length of postoperative hospital stay was 27.3 ± 21.4 days in the TXA group, compared to 33.4 ± 16.3 days in the non-TXA group. Postoperative length of hospital stay was 18.5 ± 14.8 days in the non-TXA group, compared to 20.3 ± 15.9 days in the TXA group. Our study found that, among those requiring blood transfusion, the TXA group had a 1.1 g/dL lesser Hb drop compared to the non-TXA group, despite having a lower preoperative Hb level (Table 3).

**Table 3:** Blood transfusion

Patient characteristics		
	Non-TXA (n=4)	TXA (n=3)
Sex female/male, N (%)	3/1 (75/25)	3/0 (100/0)
Mean age (SD)	80.5 (1.9)	76.3 (3.5)
Mean pre-op Hb (SD)	10.7 (0.3)	10.3 (1.3)
Mean POD1 Hb (SD)	7.9 (0.3)	8.7 (2.2)
Mean Hb drop (SD)	2.8 (0.0)	1.7 (2.3)
Mean length of postoperative hospital stay (SD)	18.5 (14.8)	20.3 (15.9)
Mean length of total hospital stay (SD)	33.5 (16.3)	27.3 (21.4)
Hb = hemoglobin, SD = standard deviation, POD = postoperative day		

## Discussion

In this single-center retrospective study, we evaluated the effects of TXA on Hb levels from preoperative to postoperative and the rate of blood transfusion following TXA administration on trauma patients undergoing RSA for PHF. Our results demonstrated that there was no significant difference in pre- and post-operative Hb between TXA and non-TXA groups (Table 2). Our findings contradict previous studies reporting significant differences in Hb postoperatively between TXA and non-TXA groups [20,21]. The contradicting results could be attributed to different cohorts between the studies. Our study involved trauma patients who are already at a greater risk of blood loss whereas the other studies have elective patients undergoing RSA.

Furthermore, contradictory to several studies, our results did not identify a significant drop in Hb levels postoperatively between the two groups (Table 2) [22,23]. Although Cuff et al. administered 1 g TXA preoperatively similar to our study, their RSA cohort comprised a smaller number of 44 patients compared to our study with 78 patients in total. Additionally, Beyth et al. used 1 g TXA topically to the shoulder joint after wound closure whereas 1 g TXA injection was administered intravenously during anesthetics induction in our study. Topical and systemic administration of TXA was previously demonstrated to be comparable; therefore, IV injection of 1 g of TXA was the chosen method of administering TXA in our service [24]. Taking into consideration that our study is the first study to our knowledge to have a greater sample of PHF cohort undergoing RSA, it could be assumed that our results were able to capture more reliable effects of IV TXA on this cohort.

Patients undergoing RSA were notably at a greater risk of blood transfusion in the elective cohort [25,26]. In this study, seven out of 78 patients (8.9%) required blood transfusion (non-TXA: 4 vs. TXA group: 3). Similar findings were also evident in previous studies whereby patients undergoing RSA received blood transfusion despite having TXA. Yoon et al. reported four out of 34 patients receiving IV TXA required blood transfusion postoperatively [24]. Likewise, Beyth et al. identified four of 32 patients receiving topical TXA in a fracture cohort needed a blood transfusion after undergoing RSA [23]. 

One study suggested pre-operative Hb lower than 11 g/dL to be a strong predictor of post-shoulder arthroplasty blood transfusion [26]. Interestingly, our study identified that among those requiring blood transfusion, despite having a lower pre-Hb level than the non-TXA group, the TXA group had a 1.1 g/dL lesser Hb drop compared to the non-TXA group (Table 3). It could be argued that TXA might possibly be aiding patients to preserve their Hb postoperatively to a certain extent. However, 1 g IV injection TXA might be a lower dose for such trauma patients who are already predisposed to greater blood loss compared to the elective cohort. Studies have shown that 2 g of IV TXA safely reduces blood loss, causes a lower Hb drop, and results in a lesser blood transfusion rate postoperatively in elective shoulder arthroplasty [18,19,27]. Further studies investigating the efficacy of different dosages of IV TXA administration on trauma patients should be carried out to ascertain whether the significant beneficial effects of IV TXA on trauma patients are dose-dependent.

Greater risk of blood transfusion following RSA has been associated with several other factors such as older age and female gender [14,26]. Our study showed that 78% of those requiring postoperative transfusion following shoulder arthroplasty were females. Our results are comparable to studies suggesting female gender is an independent risk factor for blood transfusion postoperatively [14,28]. Elderly females are at a greater risk of osteoporosis compared to males which in turn puts them at a higher risk of fragility fractures [29]. Our study provides further evidence that female patients and those with lower preoperative Hb in trauma settings are at a greater risk of blood transfusion postoperatively despite receiving TXA.

Blood transfusion is known to be associated with complications such as respiratory infections, longer hospital stays, and cardiopulmonary events [13,30]. Patients in the TXA group interestingly had a longer length of hospital stay postoperatively compared to the non-TXA group (Table 3). Longer length of hospital stay was required for treatment for hospital-acquired pneumonia, one of the most common complications following blood transfusion. Patients were also noted to have undergone physical deconditioning requiring longer inpatient physiotherapy which in turn prolonged their length of hospital stay. Blood transfusion also comes with financial implications. According to the NHS Blood and Transplant 2023/24 price list, standard red cell costs £158.18 [31]. Likewise, general ward cost per bed day was reported to be £586.59 in 2016-17. On the other hand, the median dose of IV TXA costs £1.54 as per NICE guidelines [32]. To summarize, the cost-effectiveness of using TXA in trauma cohorts undergoing RSA is yet to be investigated.

There are several strengths to our study. QEH is a major trauma center with highly skilled surgeons. It should be taken into consideration that this hospital receives complex fractures from all over the UK introducing additional challenges and financial burden on the services. Further studies should be conducted on a larger scale with the involvement of multiple centers. Our study consisted of a larger number of trauma patients compared to previous studies involving fracture cohorts undergoing RSA [22,23]. Patients in this study were predominantly females (74%) compared to 26% male patients, which reflects the higher prevalence of shoulder fractures seen in females than males [33].

On the contrary, one of the limitations of our study is that only Hb levels were assessed to investigate the efficacy of TXA in trauma patients. Assessing Hb standalone does not account for any hemodilution for surgical patients who routinely receive IV fluids perioperatively [34]. Therefore, outcome measures such as hematocrit and blood loss should be included alongside Hb levels in future studies. Likewise, the majority of our patients were elderly patients who may have multiple co-morbidities. Patients with preoperative co-morbidities are at a greater risk of poor recovery postoperatively and common complications including chest infection and heart failure [35,36]. Future studies should consider profiling patients according to their co-morbidities to take into account the effects of co-morbidities on postoperative complications following TXA administration in trauma patients.

## Conclusions

An intravenous dose of 1 g TXA preoperatively in trauma patients undergoing shoulder arthroplasty does not cause significant Hb level change from preoperative to postoperative compared to those who do not receive TXA. Future studies should consider assessing the dose-dependent efficacy of TXA in trauma patients undergoing reverse shoulder arthroplasty (RSA). Preoperative Hb and female gender remain good predictors of risk of postoperative transfusion even in the trauma cohort. TXA should still be considered for trauma patients at high risk of blood transfusion especially if they are older female patients undergoing RSA.
